# Supplementary material for: Development of a gait speed estimation model for healthy older adults using a single inertial measurement unit
Source: PLoS One. 2022 Oct 6;17(10):e0275612. doi: 10.1371/journal.pone.0275612 (PMC9536628; doi:10.1371/journal.pone.0275612)
Supplement: Attachment — Submitted filename: Response to Reviewers.docx [file pone.0275612.s001.docx]

Dear Editor,

# Thank you for your comments on our work titled as "Development of a gait speed estimation model for healthy older adults using a single inertial measurement unit”.

# Our answers to the comments that the reviewers proposed are listed in the following pages. The revisions we made are indicated in the answers and highlighted in the manuscript.

We appreciate it very much that you are considering publishing our paper in your journal.

.

Sincerely yours,

Ki Woong Kim, M.D., Ph.D.

**Responses to the reviewers' comments**

**[Reviewer #1]**

1. At the beginning of the whole study, the author needs to present your work as less word as you can, which include purpose, methods, results and conclusion. During the abstract part, it cannot catch the reader attention at the first time. Please rewrite this part.

**(Answer)**

Thank you for the comment. We totally agree with your comment and revised the abstract accordingly (PAGE 2 LINE 23-41).

2. Please further prove why this study is important. We have to confirm that this research is meaningful, rather than there is no such study that has been done yet. Author needs to well-introduce what kind of problems or issues would be solved after this research.

**(Answer)**

Thank you for the comment. We extensively revised the introduction section accordingly (PAGE 3-4 LINE 46 - 78).

3. Please make your methods part more specific with some test images and make your method more specific as well. This short explanation cannot easily understand.

**(Answer)**

Thank you for the comment. We did not describe the details of gait analysis in the method section because they are quite heavy and described in detail in our previous work cited in the current manuscript. However, for the better understanding of the readers, we added a brief summary on it with a figure (Figure 1) in the revised manuscript (PAGE 9-10 LINE 140-168 ) .

4. Did the author perform Shapiro-Wilk normality tests?

**(Answer)**

Thank you for your comment. We performed the Shapiro-Wilk normality test on the gait speed acquired from the GAITrite^TM^. P value was 0.159 in men and 0.203 in women, indicating that the gait speed was normally distributed.

**[Reviewer #2]**

1. The abstract is not clear. The significance and challenges should be presented in the abstract.

**(Answer)**

Thank you for the comment. We totally agree with your comment and revised the abstract accordingly (PAGE 2 LINE 23-41).

2. The motivations should be strengthened when revising in the introduction. The authors should further enlarge Introduction with current results to improve the research background.

**(Answer)**

Thank you for the comment. We extensively revised the introduction section accordingly (PAGE 3-4 LINE 46 - 78).

3. What are the limitations of the proposed gait estimation method? The author did not mention it in the paper.

**(Answer)**

Thank you for the comment. We described the limitations of our methods in the discussion section in more detail (PAGE 19 – 20 LINE 320-336 ).

4. It would be nice to discuss the following papers in the paper:
   - Zhang, M., Wang, Q., Liu, D., Zhao, B., Tang, J., & Sun, J. Real-time gait phase recognition based on time domain features of multi-MEMS inertial sensors. IEEE Transactions on Instrumentation and Measurement, 2021,70, 1-12.
   - Marta, G., Alessandra, P., Simona, F., Andrea, C., Dario, B., Stefano, S., Stefano, M.. Wearable Biofeedback Suit to Promote and Monitor Aquatic Exercises: A Feasibility Study. IEEE Transactions on Instrumentation and Measurement, 2020, 69(4), 1219-1231.
   - S. Qiu, H. Zhao, N. Jiang, D. Wu, G. Song, H. Zhao, Z. Wang. Sensor network oriented human motion capture via wearable intelligent system, International Journal of Intelligent Systems, 2022, 37(2): 1646-1673.

**(Answer)**

Thank you for the recommendation. The limitation of the gait speed estimation using a single sensor can be overcome by employing the multiple sensors and sensor network system. We point out this in the discussion section and cited the references you recommended. (PAGE 20 LINE 325-328)

5. Transitions from section to section should be smoother

**(Answer)**

Thank you for the comment. We tried to revise the transitions smoother in the introduction, methods and discussion accordingly.

6. What are the implications of the findings? More discussion should be provided in the manuscript. The factors that influence the accuracy of gait estimation should be analyzed in more detail in the discussion section.

**(Answer)**

Thank you for the comment. We added the implications of our model in the discussion section (PAGE 20 LINE 332-336). We discussed the factors such as walking pattern and step length that improved the accuracy of gait speed estimation in our new models (PAGE 18-19 LINE 288-319). In addition, we added a brief discussion on the misplacement of the sensor which might have also influence the accuracy of gait speed estimation (PAGE 20 LINE 328-331).

7. How do you deal with inertial sensor misplacement?

**(Answer)**

Thank you for the comment. As we described in the method section, we fixed an IMU to each participant at the 3^rd^ – 4^th^ lumbar vertebrae using Hypafix which is the soft, stretchable non-woven polyester material adapts well to body contours. We revised the method section in more detail and added the possibility of misplacement error as an additional limitation of our study (PAGE 8 LINE 129-130, PAGE 20 LINE 328-331).

8. Some individuals are not willing to wear additional sensors on their body, what is the potential of smartphone serving as the data collection tool?

**(Answer)**

Thank you for the comment. We added a discussion on the potential application of our model to a smartphone which can be employed as an inertia sensor as well as an acceleration signal processor. (PAGE 20 LINE 332-336).

9. Proofread the paper and improve readability.

**(Answer)**

Thank you for the comment. We got a proofread of the revised manuscript again from a professional proofreading service.
